# Editorial: New insights into the role of mitochondria in muscle pathophysiology

**DOI:** 10.3389/fphys.2023.1151120

**Published:** 2023-02-07

**Authors:** Oh Sung Kwon, Yuki Tamura, Yuho Kim

**Affiliations:** ^1^ Department of Kinesiology, Storrs, CT, United States; ^2^ UConn Center on Aging and Department of Orthopedic Surgery, University of Connecticut, Storrs, CT, United States; ^3^ Graduate School of Health and Sport Science, Nippon Sport Science University, Tokyo, Japan; ^4^ Department of Physical Therapy and Kinesiology, University of Massachusetts Lowell, Lowell, MA, United States

**Keywords:** mitochondria, skeletal muscle, FoxO, HIIT, lactate, L-kynurenine

## Introduction

Over the last decades there has been a big progress in defining roles of mitochondria in the cell. However, there are still unsolved questions as to how these organelles alter their molecular and functional characteristics under physiological and pathological challenges, particularly in skeletal muscle where mitochondria are key to metabolism, movement, and body temperature. Here, this Research Topic was designed to provide new insights into the roles of mitochondria in muscle physiology and had a great opportunity to collect prominent research articles that, using animal and cell models, demonstrate underlying molecular events important for mitochondrial oxidative metabolism in skeletal muscle at various physiological and pathological conditions (i.e., diabetes (Bhardwaj et al.), high-intensity interval training (HIIT) (Delfan et al.), lactate overload (Takahashi et al.), and muscle degeneration (Palzkill et al.).

## Key findings

1. Insulin plays a crucial role for mitochondrial function through interacting with insulin receptor (IR) and IGF-1 receptor (Taniguchi et al., 2006), yet the underlying mechanism is obscure especially in skeletal muscle. Using double knockout mouse model (e.g., co-deletion of IR and IGF-1 receptor), Bhardwaj et al. showed that the absence of both IR and IGF-1 receptor leads to mitochondrial dysfunction in skeletal muscle and it is related with significant changes in muscle transcriptome. Notably, they revealed that these transcriptomic regulations are dependent on the transcription factor Forkhead box O proteins (FoxOs), as mitochondria-specific molecular changes (e.g., TCA cycle genes) are normalized in the absence of FoxOs. Furthermore, this study also pointed out a critical role of FoxOs in the regulation of genes for muscle calcium signaling pathways (e.g., sarcoplasmic reticulum, transcriptional regulation, and muscle contraction). Collectively, it is likely that FoxOs are central players for the insulin action for mitochondrial capacity, as well as calcium handling in skeletal muscle.
2. Using high-intensity interval training (HIIT) model, Delfan et al. showed that this time-efficient exercise training protocol is capable of increasing mitochondrial biogenesis markers (e.g., PGC-1α, Citrate Synthase, and p53) in the skeletal muscle of diabetic animal model (i.e., streptozotocin-injected Wistar rats). In this study, they suggested that HIIT training with a higher work-to-rest ratio (HIIT with long recovery exercise; 2 min of high intensity running followed by low-intensity recovery exercise for 2 min) results in more significant increase in mitochondrial aerobic capacity in the diabetic soleus muscles as compared to the ones with a lower work-to-rest ratio (HIIT with short recovery exercise; 2 min of high intensity running followed by low-intensity recovery exercise for 1 min). Therefore, when applying HIIT, the interval length may be considered in order to improve muscle aerobic capacity.
3. Takahashi et al. suggested that lactate is a potent stimulator for upregulating mitochondrial respiratory capacity in skeletal muscle. Following long-term administration (4 weeks) of lactate *via* intraperitoneal injections, they observed a significant increase in mitochondrial oxidative capacity (e.g., state three oxygen consumption rate) in mouse skeletal muscle (i.e., gastrocnemius). In particular, it appears that these lactate-induced mitochondrial adaptations in skeletal muscle can be achieved by enhanced enzyme activity and abundance for mitochondrial electron transport chain complex I. Thus, the outcomes of this study indicate that high intensity exercise training can improve mitochondrial oxidative metabolism in skeletal muscle through activating lactate production.
4. Given that L-Kynurenine (L-Kyn), byproduct of tryptophan metabolism, is related with muscle weakness, atrophy, and neuromuscular dysfunction, Palzkill et al. sought to understand how the elevated L-Kyn affects mitochondrial function in skeletal muscle. Following 10 weeks of dietary treatment with L-Kyn (150 mg/kg), they observed that, although muscle size and contractile function are not changed, mitochondrial respiratory capacity (e.g., mitochondrial oxygen consumption) is significantly diminished in mouse skeletal muscle. Based on upregulated mRNA level of PGC-1α, this study suggests that upon elevated L-Kyn, PGC-1α may exert a compensatory response in that skeletal muscle. Using *in vitro* model, they further demonstrated that lentiviral-mediated PGC-1α overexpression has a significant effect on preserving mitochondrial function, as well as myotube structure, suggesting a possible protective role of PGC-1α against L-kyn-related muscle mitochondrial dysfunction.

## Closing comments

In this Research Topic, we can pull out several key markers for mitochondrial function, which is valuable to find possible therapeutic targets for maintaining or improving muscle health. Furthermore, these findings can be further developed to understand how those molecular systems are linked with other mitochondrial control systems such as mitochondrial turnover (fusion/fission), quality control (mitophagy), and structural changes in skeletal muscle.
